# Gendered achievement patterns in undergraduate education: evidence from a bachelor’s program

**DOI:** 10.1186/s13104-026-07788-5

**Published:** 2026-03-28

**Authors:** Marius Ole Johansen, Kjersti Elisabet Lea, Helene Marie Kjærgård Eide

**Affiliations:** Department of Education, University of Bergen, Bergen, Norway

**Keywords:** Gender differences, Grade distribution, Higher education

## Abstract

**Background:**

Responding to the broader call for transparency and accessibility in educational data analysis, this research note presents a concise descriptive analysis of cumulative grade distributions for male and female students across a decade.

**Objective:**

The objective of the research presented is to document persistence in gender difference in student performance at a bachelor’s program in pedagogy over time.

**Results:**

Spanning a decade, the descriptive analysis highlights persistent differences between male and female students in cumulative grade distribution. Across the entire grade scale, the cumulative grade distribution for students indicates systematically higher grades among females and reflects a higher concentration of top grades among female candidates.

## Introduction

Since the 1970s, the rise of data in education, or so-called governing by numbers, has made national repositories for administrative data about the population the key knowledge base for governing and evaluation of public services in most Western countries [1]. The Database for Statistics on Higher Education (DBH) is Norway’s central repository for administrative data on students, study programs, and assessment outcomes across all accredited higher education institutions [2]. As part of national quality assurance efforts, universities and colleges annually report detailed information on course completion, grades, and progression patterns [3]. These data are publicly accessible and provide a unique opportunity not only to make governing choices, but also to describe long-term trends in educational outcomes across demographic groups. Despite the richness of DBH, national administrative data are rarely used to present simple, transparent descriptive analyses of grade distributions over time, particularly using cumulative probability representations to examine demographic differences. Further, while demographic disparities in educational outcomes are a well-recognized concern, longitudinal and population-level evidence, particularly in higher education, remains relatively limited, with most existing work focusing on earlier stages of schooling or institution-specific samples [4].

Differences in males’ and females’ academic performance have been documented across several educational systems, including the Nordic countries [5, 6]. Reports from secondary and upper-secondary education consistently show that female students obtain higher grades than male students (see, e.g. 7–11). Although female grade advantages are commonly observed in secondary and upper-secondary education [12], findings from tertiary education are less uniform and depend on field of study, assessment practices, and institutional context [13]. This calls for transparent, descriptive analyses of national administrative data to assess whether such differences persist. Much of this literature on sex differences in academic performance, however, relies on institution-specific datasets or focuses on single programs or cohorts, making it challenging to obtain a national overview of long-term trends. Administrative datasets like DBH could fill this gap, yet their descriptive potential is rarely utilized in the published literature. The present study originates from an exploratory effort to better understand grade distributions at the bachelor level in Norway and, in particular, whether gender differences observed persist consistently.

This research note presents a concise descriptive analysis of 10 years of bachelor grade data, disaggregated by sex and visualized using cumulative distribution curves. The aim of this contribution is not to provide causal explanations or theoretical interpretations of differences in higher education performance. Rather, the objective is to document a simple, robust empirical observation derived from public administrative sources. By presenting the cumulative grade distributions for male and female students across a decade, we highlight a pattern that appears consistent and stable over time and that may be of interest to researchers, policymakers, and institutions seeking to understand broader performance trends in Norwegian higher education. The study also responds to the broader call for transparency and accessibility in educational data analysis [14]. Descriptive statistics derived from national administrative data may be used to identify persistent or systematic differences in educational outcomes, for example across demographic categories such as sex. As such, analyses of this kind may support institutional quality work by providing empirical basis for monitoring patterns and development over time and identifying areas warranting further investigation.

## Materials and methods

### Data source and extraction

This study builds on data obtained from the Database for Statistics on Higher Education [2] in Norway. DBH provides aggregated statistics that are publicly available via the DBH public statistics portal. For this analysis, we extracted aggregated grade-counts for bachelor degrees in pedagogy awarded at one of the country’s largest universities. The extraction covered the most recent ten-year span available at the time of data retrieval. The extracted dataset contains the following variables: academic year (year in which the assessment was completed), registered sex, grade category: A (highest), B, C, D, E, F (lowest; denotes fail), and the count of awarded grades for each year × sex × grade combination. Inclusion criteria were limited to bachelor-level degrees in pedagogy at this particular university.

No individual-level records or personal identifiers were accessed. The DBH export used here provided complete annual grade counts for the included years and program; where DBH suppresses or rounds very small counts according to its public reporting rules these cases are acknowledged as possible but did not affect the primary cumulative-distribution comparisons.

## Results

**Fig. 1:**
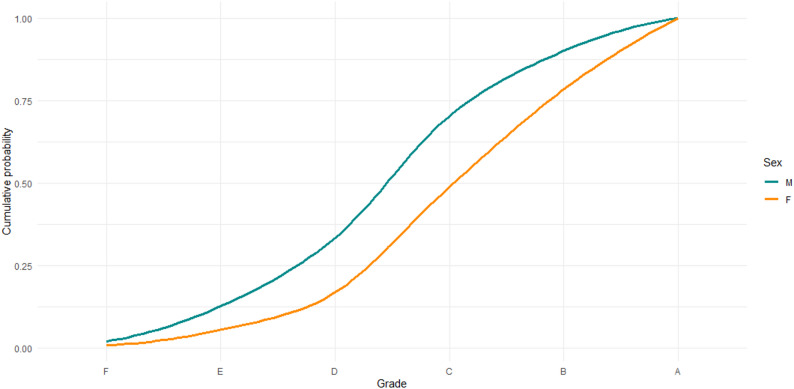
Cumulative grade probabilities for bachelor theses stratified by sex

Figure 1 displays cumulative grade probabilities for bachelor theses in pedagogy, stratified by sex (*N* = 924). The curves represent the proportion of students who have achieved a given grade or lower. Lower cumulative probabilities at a given grade threshold reflect higher overall academic performance. Across the entire grade scale, the cumulative distribution for female students is consistently shifted to the right relative to that of male students, indicating systematically higher grades among females. For example, at grade C, approximately 50% of female students have received this grade or lower, compared to roughly 70% of male students. Conversely, this implies that about half of female students obtained a grade higher than C, whereas only around 30% of male students did so. Similar differences are observed at higher grade thresholds. At grade B, approximately 80% of female students have achieved B or lower, compared to close to 90% of male students, again reflecting a higher concentration of top grades among female students.

These patterns persist across the full distribution, suggesting that the observed difference is not driven by extreme outcomes but reflects a general shift in grade distributions. Overall, the cumulative probability based on these data highlights clear and systematic differences in bachelor’s thesis grades between female and male students, offering a transparent illustration of performance disparities that are not readily captured by mean grades alone.

**Fig. 2:**
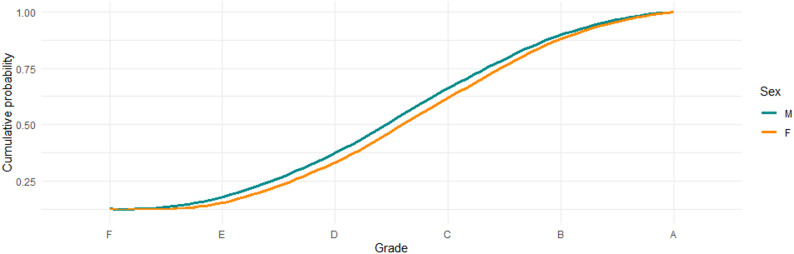
Cumulative grade probabilities for a course using written assessment exam stratified by sex

To contextualize these findings, we conducted a parallel descriptive analysis for a core pedagogy course assessed by a six-hour written school examination. Figure 2 displays the cumulative grade probabilities for this course, stratified by gender. In contrast to the bachelor thesis grades, the cumulative distributions for male and female students are nearly overlapping across the entire grade scale. Differences at specific grade thresholds are small and inconsistent, generally on the order of 2–3% points. This indicates that the pronounced differences observed for bachelor thesis grades are not mirrored in this written examination context. These contrasting patterns indicate that the differences in grading outcomes are not uniform across assessment formats, highlighting a need for further research to examine how assessment context may relate to observed performance differences.

The present study intentionally focuses on a single bachelor’s program at one institution to ensure contextual consistency in teaching practices, assessment formats, grading cultures, and curricular implementation. Broadening the analysis to multiple institutions, disciplines, or national aggregates would introduce substantial heterogeneity that would complicate the interpretation of gender differences in grade distributions for this research note. Rather than making claims about gender differences across Norway or across disciplines, the primary purpose is to demonstrate the utility of publicly available administrative data and to provide a transparent, reproducible analytical workflow. To facilitate broader use, all the analysis code is openly available, enabling researchers to readily extend the approach to other programs, institutions, disciplines, or national datasets. In this sense, the present analysis serves as a template for future descriptive investigations using available administrative education data.

## Limitations

This study is subject to several limitations inherent to the use of aggregated administrative data and to its purely descriptive scope. First, the DBH data used here are reported in aggregated form, which precludes analyses at the individual level and prevents adjustment for potentially relevant covariates such as prior academic achievement, age, enrollment status, or socioeconomic background. As a result, the observed differences in grade distributions should not be interpreted as causal effects, nor as reflecting inherent or predetermined differences between male and female students. Second, the analysis is restricted to bachelor’s degrees in pedagogy awarded at a single Norwegian institution. Although this university is a large, research-intensive university, the extent to which the observed patterns generalize to other institutions, disciplines, or national contexts cannot be determined from the present data. Third, grades are treated as ordinal outcomes and summarized using cumulative distributions. While this approach provides a transparent and assumption-light comparison across groups, it does not capture potential differences in grading practices, assessment formats, or cohort composition over time. Further, grading practices and assessment contexts may change over time, which should be considered when interpreting the descriptive patterns reported here. Finally, DBH applies rounding and suppression rules for very small cell counts in its public reporting, which may introduce minor imprecision in some grade categories, although this is unlikely to affect the overall cumulative distribution patterns presented.

## Data Availability

Data were obtained from the Database for Statistics on Higher Education (DBH), available at https:/dbh.hkdir.no/tall-og-statistikk. The dataset used in this study consists of student statistics from the University of Bergen, Faculty of Psychology, Bachelor’s program in Pedagogy. The data can be retrieved by navigating within the DBH portal to the relevant study program and selecting the specified variables. A direct query link is available from the authors upon request. The code used to reproduce the analyses is available at (https:/github.com/MariusJohans/s_24).

## Abbreviations

DBH: The Database for Statistics on Higher Education
